# Chimeric Hepatitis B core virus-like particles harboring SARS-CoV2 epitope elicit a humoral immune response in mice

**DOI:** 10.1186/s12934-023-02043-z

**Published:** 2023-02-25

**Authors:** Sima Sazegari, Malihe Akbarzadeh Niaki, Alireza Afsharifar, Ali Niazi, Abdollah Derakhshandeh, Maryam Moradi Vahdat, Farshad Hemmati, Mohammad Hadi Eskandari

**Affiliations:** 1 Institute of Biotechnology, Shiraz University, Shiraz, Fars Iran; 2 Department of Food Science and Technology, Shiraz University, Shiraz, Fars Iran; 3 Plant Virology Research Center, College of Agriculture, Shiraz University, Shiraz, Iran; 4 Department of Pathobiology, School of Veterinary Medicine, Shiraz University, Shiraz, Iran

**Keywords:** HBc VLP, Vaccine, Virus-like particle, SARS-CoV2, Recombinant expression

## Abstract

**Background:**

Virus-like particles are an interesting vector platform for vaccine development. Particularly, Hepatitis B virus core antigen has been used as a promising VLP platform. It is highly expressed in different recombinant expression systems, such as *E. coli,* and self-assembled in vitro. It effectively improves the immunogenicity of foreign antigenic epitopes on its surface. Various foreign antigens from bacteria, viruses, and protozoa can be genetically inserted into such nanoparticles. The effective immunogenicity due to VLP vaccines has been reported. However, no research has been performed on the SARS-CoV2 vaccine within this unique platform through genetic engineering. Considering the high yield of target proteins, low cost of production, and feasibility of scaling up, *E. coli* is an outstanding expression platform to develop such vaccines. Therefore, in this investigation, we planned to study and develop a unique HBc VLP-based vaccine against SARS-Cov2 utilizing the *E. coli* expression system due to its importance.

**Results:**

Insertion of the selected epitope was done into the major immunodominant region (MIR) of truncated (149 residues) hepatitis B core capsid protein. The chimeric protein was constructed in PET28a^+^ and expressed through the bacterial *E. coli* BL21 expression system. However, the protein was expressed in inclusion body forms and extracted following urea denaturation from the insoluble phase. Following the extraction, the vaccine protein was purified using Ni2 + iminodiacetic acid (IDA) affinity chromatography. SDS-PAGE and western blotting were used to confirm the protein expression. Regarding the denaturation step, the unavoidable refolding process was carried out, so that the chimeric VLP reassembled in native conformation. Based on the transmission electron microscopy (TEM) analysis, the HBC VLP was successfully assembled. Confirming the assembled chimeric VLP, we explored the immunogenic effectivity of the vaccine through mice immunization with two-dose vaccination with and without adjuvant. The utilization of adjuvant was suggested to assess the effect of adjuvant on improving the immune elicitation of chimeric VLP-based vaccine. Immunization analysis based on anti-spike specific IgG antibody showed a significant increase in antibody production in harvested serum from immunized mice with HBc-VLP harboring antigenic epitope compared to HBc-VLP- and PBS-injected mice.

**Conclusions:**

The results approved the successful production and the effectiveness of the vaccine in terms of humoral IgG antibody production. Therefore, this platform can be considered a promising strategy for developing safe and reasonable vaccines; however, more complementary immunological evaluations are needed.

## Introduction

As the SARS-CoV-2 infection has spread over the world and the pandemic expanded, the need for the development of effective preventive vaccines has launched. Owing to the advancements in sequencing and molecular characterization of the SARS-CoV-2 genome, various vaccines have been studied and developed against the virus [1]. Although the emergence of mutant variants affects the effectiveness of vaccines, vaccination is considered the most powerful strategy to control the outbreak. Because the structural spike (S) protein triggers the infection of the target cell, as the SARS-CoV-2 virus penetrates the cell via the attachment of the S protein to cellular receptors known as human angiotensin-converting enzyme 2 (ACE2), it is considered the most promising target for vaccine development against COVID-19 infection [2–4]. Nevertheless, most of the commercially developed vaccines, such as BNT162b2 of Pfizer/BioNTech and ChAdOx1 nCoV-19 of AstraZeneca were designed and constructed based on the S protein [5, 6]. Thus, the development of an effective vaccine is mainly the most protective strategy to control this infection.

Virus-like particles (VLPs), ranging from 20 to 100 nm in size, are generated by structural viral proteins and can be self-assembled. While resembling viruses, they are non-infectious because they do not have viral genetic material [7–9]. Due to their particular structure and repetitive surface patterns, they can promisingly display immunogenic epitopes on their surface and elicit specific powerful immune responses [10]. Different chimeric VLP-based vaccines against animal and human infectious diseases have been investigated in the last few years [11–15]. One of the most potent VLP candidates for packaging and displaying foreign epitopes and triggering particular immunogenicity is the hepatitis B core (HBc) VLP [16, 17]. Based on the experimental investigations, the major immunodominant region (MIR), spanning into amino acids 76–82, is known as the most effective region for antigen insertion without disrupting the assembling potential [18]. Efficient insertion durability, effective self-assembly, high yield of VLP production and efficient exposure of the antigenic epitopes, and the possibility of production in different expression systems make this platform an ideal system to develop vaccines [19–21].

Different VLP‐based vaccines against the SARS-CoV-2 utilizing varied VLP platform and diverse expression systems have been investigated [22, 23] reported the development of Chimeric VLP-Based COVID-19 Vaccine utilizing SARS-CoV-2 Spike protein and the influenza virus A matrix (M1) VLP. They used human cell lines eukaryotic expression system for vaccine production. According to their results, high titers of spike specific IgG and neutralizing antibodies were induced through vaccine injection. In another study, the RBD protein from SARS-Cov2 was chemically fused to modified HBs-Ag protein and formulated [23, 24]. The comprehensive immunization results from vaccine injection approved the high protection against SARS-CoV-2 in mice. However, no report has been published on the SARS-CoV2 vaccine within HBc platform through genetic engineering in *E.coli*. Considering the high yield of target proteins, low cost of production, and feasibility of scaling up, *E. coli* is an outstanding expression platform to develop such vaccines. Therefore, in this investigation, we aimed to study and develop a unique HBc VLP-based vaccine platform against SARS-Cov2 utilizing the *E. coli* expression system due to its importance.

Identification of conserved and efficient epitopes based on bioinformatics analysis is crucial for developing promising immunogenic vaccines. In our previous study, immunogenic epitopes based on spike protein have been studied through computational biology approaches [25]. Besides, using bioinformatics and immunoinformatic analysis, we evaluated the efficiency and safety of predicted epitopes. Here, we applied one of the characterized E29 epitopes to construct a chimeric VLP- based vaccine based on HBc protein. A chimeric vaccine protein was constructed and evaluated by inserting the E29 epitope into the MIR region of the truncated HBc VLP. Although some studies have been reported on SARS-CoV2 VLP vaccines using different VLP types and expression systems, this study is a novel empirical investigation on production of chimeric SARS-CoV-2 vaccine based on HBc VLP platform in *E. coli* expression system.

## Results

### Confirmation of recombinant construct and protein expression in *E. coli*

**Fig. 1:**
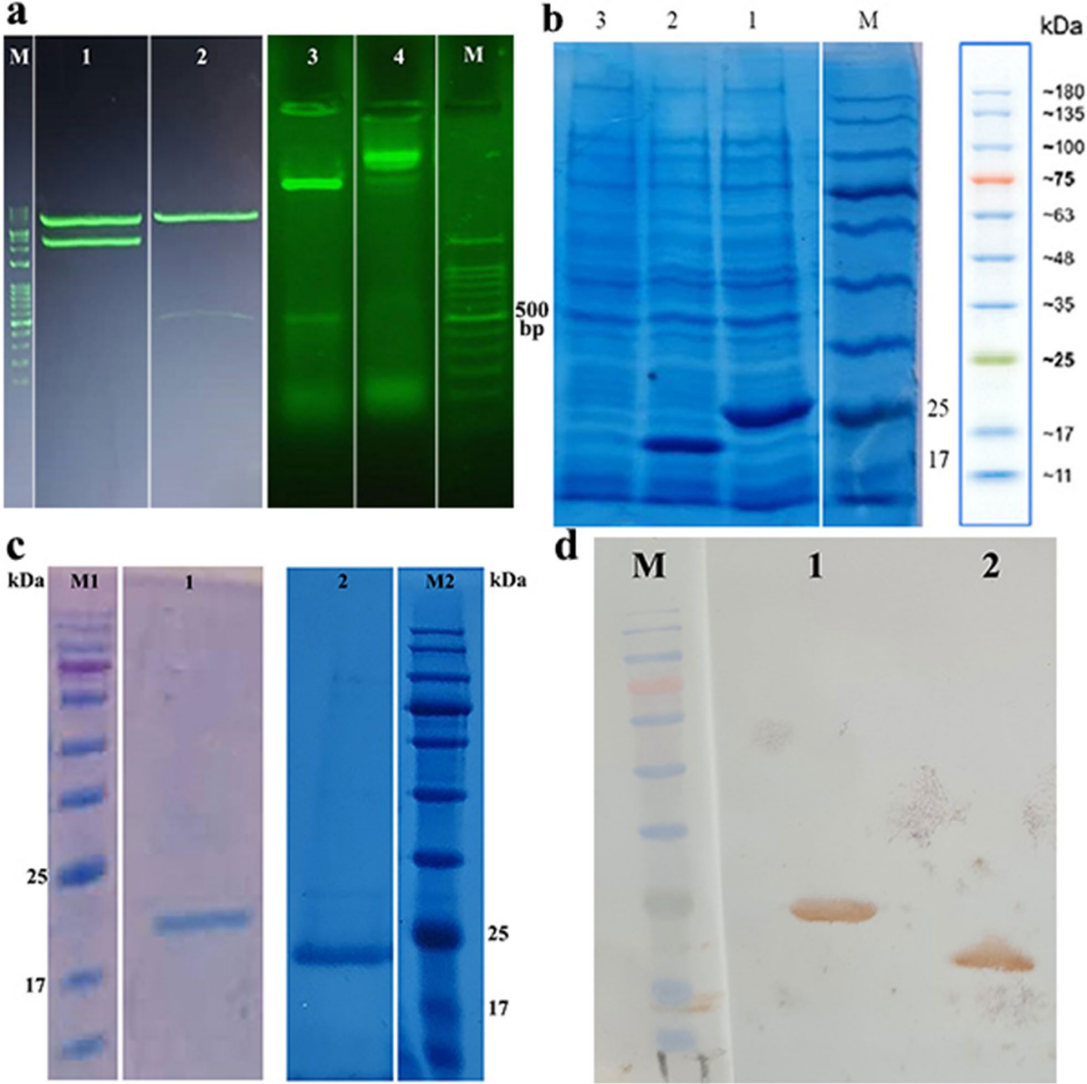
Molecular confirmation of recombinant construct and the protein expression in *E. coli.* a) Recombinant HBc VLP-E and recombinant HBc VLP plasmids after digestion by *Eco*RI and *Xho*I restriction enzymes analyzed by agarose gel electrophoresis. M) DNA ladder 100 bp, 1) undigested recombinant HBc VLP-E plasmid, 2) digested recombinant HBc VLP-E plasmid 3) digested recombinant HBc VLP plasmid 4) undigested recombinant HBc VLP plasmid **b)** SDS-PAGE analysis of the expression of HBc VLP and HBc VLP-E in *E. coli*. M) protein marker, 1) induced cells expressing HBc VLP-E on size 23.7 kDa, 3) induced cells expressing HBc VLP on size 20.3 kDa. 3) non-induced cell **c)** SDS-PAGE analysis of the purification of HBc VLP and HBc VLP-E. M1 and M2) protein marker, 1) purified HBc VLP protein, 2) purified HBc VLP-E protein **d)** Western Blot analysis of HBc VLP and HBc VLP-E proteins. M) protein marker, 1) purified HBc VLP-E protein, 2) purified HBc VLP protein

Following *E. coli* BL21 transformation, the recombinant HBc VLP and HBc VLP-E constructs were confirmed after digestion by *Eco*RI and *Xho*I restriction enzymes and agarose gel electrophoresis (Fig. 1a). Electrophoresis of plasmid digestion with selected enzymes resulted in the release of bands (453 and 546 bp) corresponded to HBc VLP and HBc VLP-E inserted from PET28a plasmid, respectively. Following the expression induction in *E. coli*, the expression of HBc VLP and HBc VLP-E were confirmed through SDS-PAGE according to the observation of the predicted protein bands (20.3 and 23.7 kDa) on the gel (Fig. 1b). Moreover, after purification of His-tagged recombinant proteins based on Ni^+^ IDA resin, they were again analyzed by SDS-PAGE to investigate the presence and purity of the purified proteins (Fig. 1c). Besides, for complementary analysis, the purified protein was confirmed by His-tag monoclonal antibody-HRP conjugated through Western blotting. The Western blot analysis approved the successful expression of HBc VLP and HBc VLP-E proteins based on recognition with anti-His antibody (Fig. 1d). In general, the results of this part confirmed the expression of the recombinant proteins in the *E. coli* expression system.

### Electron microscopy analysis of HBc VLP structure

**Fig. 2:**
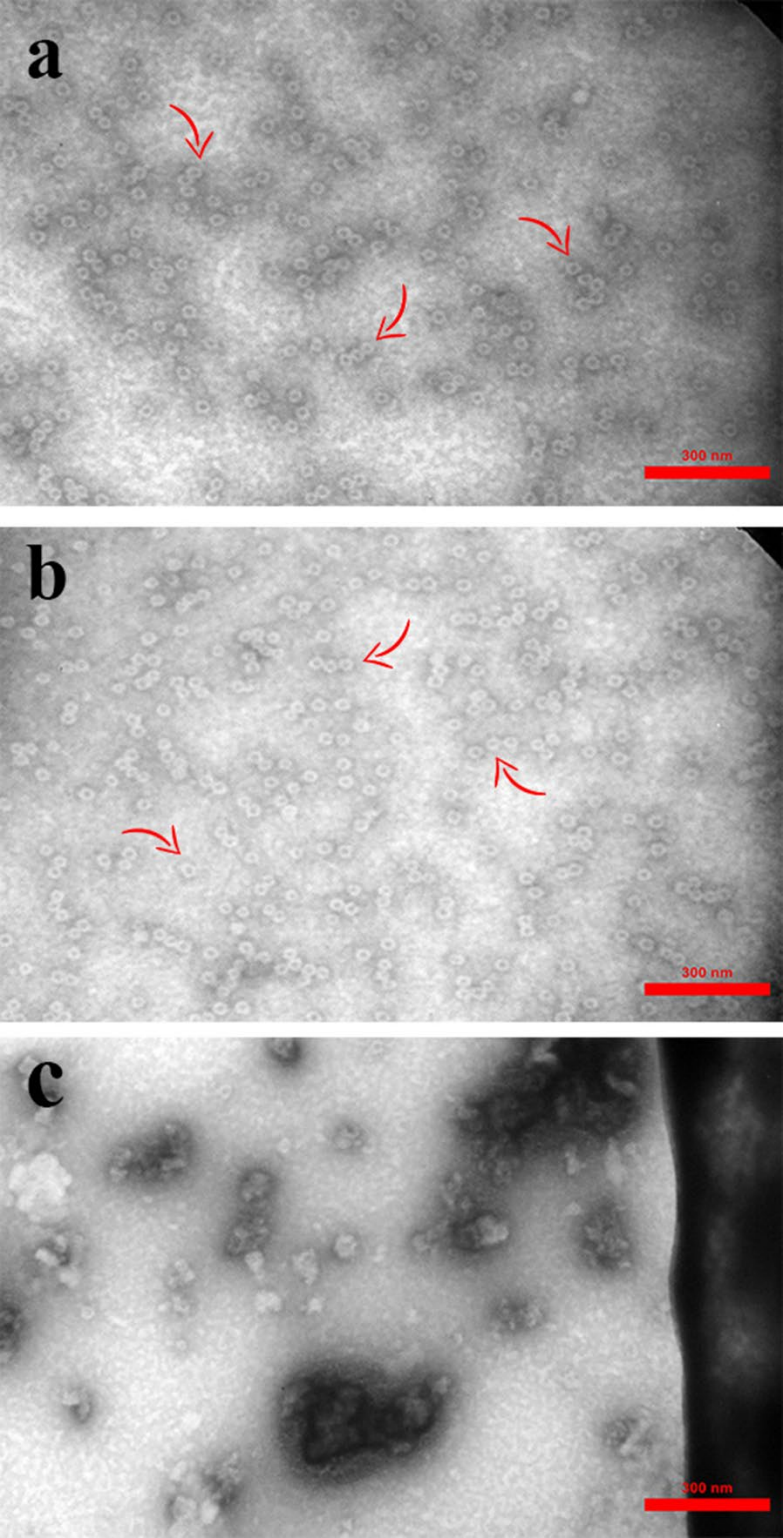
Transmission electron microscopic analysis of refolded **a** HBc VLP and **b)** HBc VLP-E proteins versus **c** the denatured proteins before the purification and refolding process. Assembled proteins are indicated by arrow. Scale bar, 300 nm

To investigate the proper reassembling of the HBc VLP and the chimeric VLP containing the spike epitope, they were analyzed using transmission electron microscopy (TEM). Based on microscopic analysis, both VLPs showed the proper conformation unique to HBc VLP structure and were confirmed (Fig. 2a, b).

### Identification of spike-specific IgG antibody in immunized mice

**Fig. 3:**
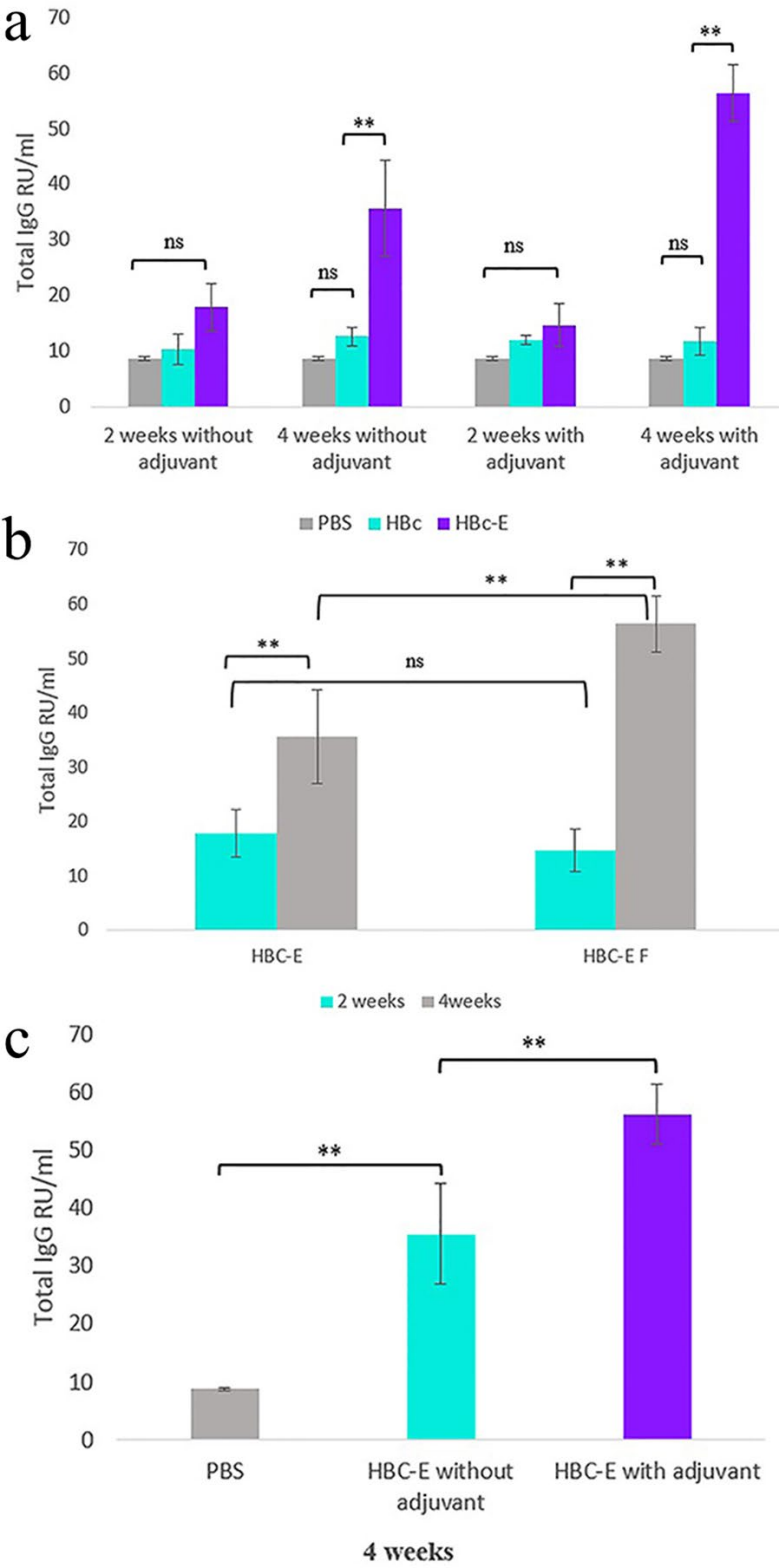
**a** Immunization analysis of mice injected with HBc VLP-E vaccine, HBc VLP, and PBS controls. The total specific SARS-CoV-2 spike IgG production presented in RU/ml two and 4 weeks after the second dose injection. Each measure is presented as the mean ± SD value of three mice with two replicates and analyzed by Duncan's test (*p* < 0.01). **b** Comparison of the specific IgG production between the two intervals after the HBc-VLP E immunization in both conditions with and without adjuvant. Each measure is presented as the mean ± SD value of three mice analyzed by Duncan’s test (*p* < 0.01). **c** Comparison of the specific IgG production with and without adjuvant utilization after four weeks of HBc-VLP E injection. Each measure is presented as the mean ± SD value of three mice with two replicates and analyzed by Duncan's test (*p* < 0.01)

Following the injections, all injected mice survived. To study the immune response resulting from the injection of chimeric HBc VLP compared to HBc VLP and PBS injected groups, serological analysis using ELISA was carried out and resulting data were statistically analyzed. The results indicated that the chimeric HBc VLP-E vaccine could stimulate the immune system, and the antibody against the antigenic epitope related to spike protein was successfully produced. Also, based on ELISA analysis, the IgG titer was significantly different in HBc VLP-E collected sera compared to HBc VLP after 4 weeks (Fig. 3a). Regarding the two different time intervals, at which the sera were harvested, the results exhibited better response of the immune system in terms of IgG production 4 weeks after the second dose injection in both conditions with and without adjuvant (Fig. 3b). The results for 4 weeks post second injection showed that in both with and without adjuvant cases, the antibody titer has been increased significantly compared to control PBS. Moreover, the with adjuvant group showed higher titer of antibody than the without adjuvant group and the statistical analysis approved the significant difference between the conditions with and without adjuvant 4 weeks after the second injection (Fig. 3c).

## Discussion

In this study, the chimeric HBc-VLP harboring the SARS-CoV-2 spike epitope was expressed in *E. coli* and the unique icosahedral conformation of HBc was confirmed. Moreover, the immunogenic potential of the investigated vaccine platform was studied in terms of IgG antibody production. The results showed the efficiency of HBc-epitope chimeric protein as a suitable vaccine candidate against SARS-CoV-2 infection; however, further structural improvements and more immunogenic evaluations are needed.

HBc VLP possesses unique properties, including successful presentation of antigenic epitopes, stability and high packaging capacity (up to 100 amino acids), and stimulating-dependent immunity, which make it a remarkable scaffold to carry and expose antigens. It is possible to insert the immunogenic epitopes in various regions of HBc, including the N-terminal, C-terminal, and MIR; however, the researchers mostly use the MIR region [26–28]. This region is known as the major insertion site with high efficiency in epitope presenting at the tips of VLP along with maintaining the self-assembling characteristic and is ideally able to trigger a vigorous immune response according to a particular antigen [29, 30]. According to the preferable features of the MIR region, we constructed the chimeric protein based on the MIR insertion type for this investigation.

Various investigations have been carried out on using HBc VLP as a vehicle for delivering antigens in vaccine development studies. Lei et al. utilized the hepatitis B virus core MIR region to present different epitopes related to the foot-and-mouth disease virus through the BL21 *E. coli* expression system [31]. Similarly, incorporating and expressing antigenic domains of *Neisseria meningitidis* into MIR and C-terminus of HBc in *E. coli* resulted in successful specific IgG stimulating and production [32]. Antigenic peptides corresponding to Enterovirus 71 (EV71) were also carried in the HBc MIR region to develop a vaccine for *E coli*. Consistent with other studies, the potential of the vaccine to induce specific immunity was reported [33, 34]. However, the expression of VLPs in the form of an inclusion body using *E. coli* expression system has been reported and remained the main struggling issue of the downstream processes, such as purification and self-assembling [8, 33, 35, 36]. Similar to these reports, we encountered the same obstacle as both HBc VLP and HBc-E VLP were mostly expressed in inclusion forms of included bodies. Hence, additional steps, including renaturing and refolding processes were performed to obtain the proper re-assembled VLP conformation. Although these challenges could affect the efficiency of epitope presentation and subsequently, hinder the proper immune response, the microscopic analysis showed that the VLPs were correctly refolded to form the HB core structure in this study.

Different expression systems, including both prokaryotic and eukaryotic systems, are applied in VLP-based vaccine development programs [37, 38]. According to the literature, bacterial and yeast expression platforms dominate other expression systems for the production of HBc VLP-based vaccines. Purification and assembling facilities are two main factors in selecting the proper expression system for the HBc VLP-based vaccine. In spite of the challenge of aggregate formations in *E. coli* expression systems, due to the high yield of expression along with cost-effectiveness, we selected this system for the production of HBc chimeric protein in this study. According to the results, the yield of purified HBc VLP and HBc-E VLP was ~ 0.4 and 0.2 mg/ml, respectively.

To evaluate the HBc-E VLP immune effect, the mice were analyzed at two different time points after injection in two conditions, including with and without adjuvant. According to the results, the adjuvant positively affected the immune response in terms of IgG antibodies at four weeks. Consistent with these results, [13] reported that the maximum antibody titer was produced when the HBc-fused antigen was injected with Freund’s adjuvant. In spite of the self-adjuvant property of the VLP vaccine, the utilization of proper adjuvant can strongly elevate the efficient immune response [13, 39, 40]. Although Freund’s adjuvant might not be safe for humans, because it is reported to be more highly immunogenic than other adjuvants in mice [41, 42], we preferably used this adjuvant to examine the general response of HBc-E VLP in the presence of a potent adjuvant in comparison with immunized mice that were solely injected with HBc-E VLP. Similarly, this adjuvant has been used in different reports due to its effectiveness in antibody production [43–46]. Moreover, as expected for the SARS-CoV-2 vaccine, in both with and without adjuvant conditions, the IgG titer was normally higher 4 weeks after injection than 2 weeks later. Similarly, mice immunization and IgG evaluation through a VLP-based developed vaccine for SARS-CoV-2 based on S1 and S2 epitopes showed that a significant amount of anti-spike IgG antibody was produced 28 days after the immunization program, during which two doses of vaccine were administered on days 0 and 21 [47]. Although the humoral immunity results indicate the immunoactivity of the recombinant protein vaccine and confirm its successful expression, introducing this antigenic protein as an efficient vaccine needs comprehensive cellular immunity evaluations. Moreover, using only one small epitope may not provide highly durable protection. Thus, considering the efficient performance of the current chimeric platform in this study, advanced constructs harboring multi-epitopes instead of just one small linear epitope along with cellular and humoral immune response assessments should be considered in future studies.

## Conclusions

According to our results, the truncated HBc protein could successfully accommodate the SARS-CoV-2 spike epitope without any disruption in self-assembling. The chimeric HBc-E VLP could also effectively elicit the immunity response in mice and induce the SARS-CoV-2 anti-spike-specific antibody. This platform can be applied as a suitable scaffold with the potential for substituting other immunogenic antigens for different pathogenic variants. Thus, developing the HBc-VLP-based vaccine for SARS-COv2 can be considered a suitable vaccine platform due to its ease of production and effectiveness.

## Material and methods

### Construction, expression, and isolation of recombinant proteins

We used the truncated form of HBc amino acids 1–149 and avoided amino acids 150 − 183 because this region includes the RNA/DNA-binding site of the viral capsid and is not necessary for the self-assembling of HBc VLP. The truncated sequence of HBc was optimized and synthesized by the GenScript company. The HBc gene was cloned into *Eco*RI and *Xho*I sites of PET28a( +) and used as a control construct, which represented as HBc VLP. Similarly, HBc-E chimeric protein composed of truncated HBc, and the E29 epitope contained nine residues (RLNEVAKNL), flanked with two G4SG4 linkers, and was inserted into the MIR region of HBc between amino acids 78 and 79. The designed construct was optimized, synthesized, and cloned at the same position as HBc VLP in PET28a ( +) and named as HBc VLP-E construct. The *Escherichia coli* BL21(DE3) cells were transformed with the plasmids using the electroporation method. A transformed colony cultured on solid LB-kanamycin (50 mg/l) was used for liquid culture in 5 ml LB media at 37 °C, followed by incubation for 16 h. To induce the expression of proteins, the overnight cultures were diluted with 250 ml of LB media containing 50 μg/ml kanamycin and grown at 37 °C to reach OD600 0.7–0.8. The induction of recombinant protein expression was through the addition of isopropyl-β-thiogalactopyranoside (IPTG) to a final concentration of 0.1 mM at 25 °C for additional 16–18 h. The cells were harvested by centrifuge of culture fluid at 4.000 ×g for 20 min. For the isolation of VLPs predominantly formed in inclusion bodies, we used the method developed by Bin Mohamed [48] with some modifications [48]. In detail, the pellets from centrifugation were re-suspended in the 15 ml of lysis buffer, including (0.2% v/v Triton X-100, 50 mM Tris, 5 mM EDTA, and 100 mM NaCl, pH 8.0). To complete the cell disruption, the cells were frozen and thawed at − 80 °C/37 °C for eight cycles. Afterward, the cell lysate was centrifuged at 18,000 g at 4 °C for 30 min. The cycle was repeated one more time to wash the cell efficiently. Because the inclusion bodies were the predominant form of the expressed proteins, the pellets were denatured by keeping them in 20 ml of denaturing buffer overnight at 4 °C. The pH of denaturing buffer made by 200 mM NaCl, 4 M urea, 10 mM 2-mercaptoethanol, and 50 mM sodium carbonate was adjusted to 9.5 before use. The next day, the denatured buffer was re-centrifuged at 18,000 g for 30 min at 4 °C and the supernatant containing the HBc VLPs was collected.

### Purification and reassembling of recombinant proteins

For purifying the HBc VLP and the HBc VLP-E chimeric proteins, we used Ni2 + iminodiacetic acid (IDA) affinity chromatography because the proteins contain N-terminal His-tag due to the utilization of PET28a vector for protein expression. Based on the method introduced by Bin Mohamed [48], a 20 ml Ni–NTA Sepharose column was packed with 3 ml of Ni–NTA resin slurry and then equilibrated with a 3 × volume of denaturing buffer. Then, the protein was transferred into equilibrated resin while the column was capped tightly at both ends by parafilm and incubated for 30 min for improved attachment. Following the removal of flow-through, the column containing His-tagged recombinant proteins was washed with 3 × volume of denaturing buffer. Finally, the bounded recombinant proteins were washed using 7 ml of elution buffer (pH 9.5), containing 200 mM NaCl, 4 M urea, 10 mM 2-mercaptoethanol, 50 mM sodium carbonate, and 1 M imidazole [48]. The concentration of washed fractions was measured using the Bradford method and analyzed by sodium dodecyl sulfate–polyacrylamide gel electrophoresis (SDS-PAGE). Since the proteins were denatured following denaturing with 4 M urea, the purified protein was subjected to refolding and assembly process. For efficient re-assembling of VLPs, purified proteins were dialyzed in a dialysis tube with 10 K MWCO using dialysis tubing against 2L of dialyzing buffer with pH 7, containing 50 mM Tris, 500 mM NaCl, and 0.5 mM EDTA.

### Evaluation of recombinant proteins using SDS-PAGE and Western blotting

Confirmation of recombinant protein expression was carried out by SDS-PAGE and Western blotting. For Western blotting, the purified His-tagged proteins were transferred from 12% polyacrylamide gel to nitrocellulose paper through semi-dry electroblotting. The membrane was then blocked by skim milk overnight followed by incubation with anti-His antibody − HRP conjugate. The membrane was washed by PBST buffer, followed by staining with (3,3′-Diaminobenzidine) DAB chromogen, and subsequently, exposed to hydrogen peroxide to detect the recombinant proteins. The sites of HRP-bound antibodies corresponding to His-tagged recombinant protein appeared in brown bands and were confirmed [49].

### Assessment of HBc VLP and HBc VLP-E using electron microscopy

The re-assembling of the target proteins was confirmed by TEM analysis. The purified refolded proteins, including HBc VLP-E and HBc VLP, were linked to 200 mesh formvar-carbon-coated copper grids and stained negatively using 1% uranyl acetate aqueous solution for 2 min. Besides, the purified protein fractions that were not conducted in the downstream refolding process (before dialysis), were used as controls. A transmission electron microscope was used to visualize VLPs [50].

### Mouse immunization with recombinant protein vaccines

**Fig. 4:**
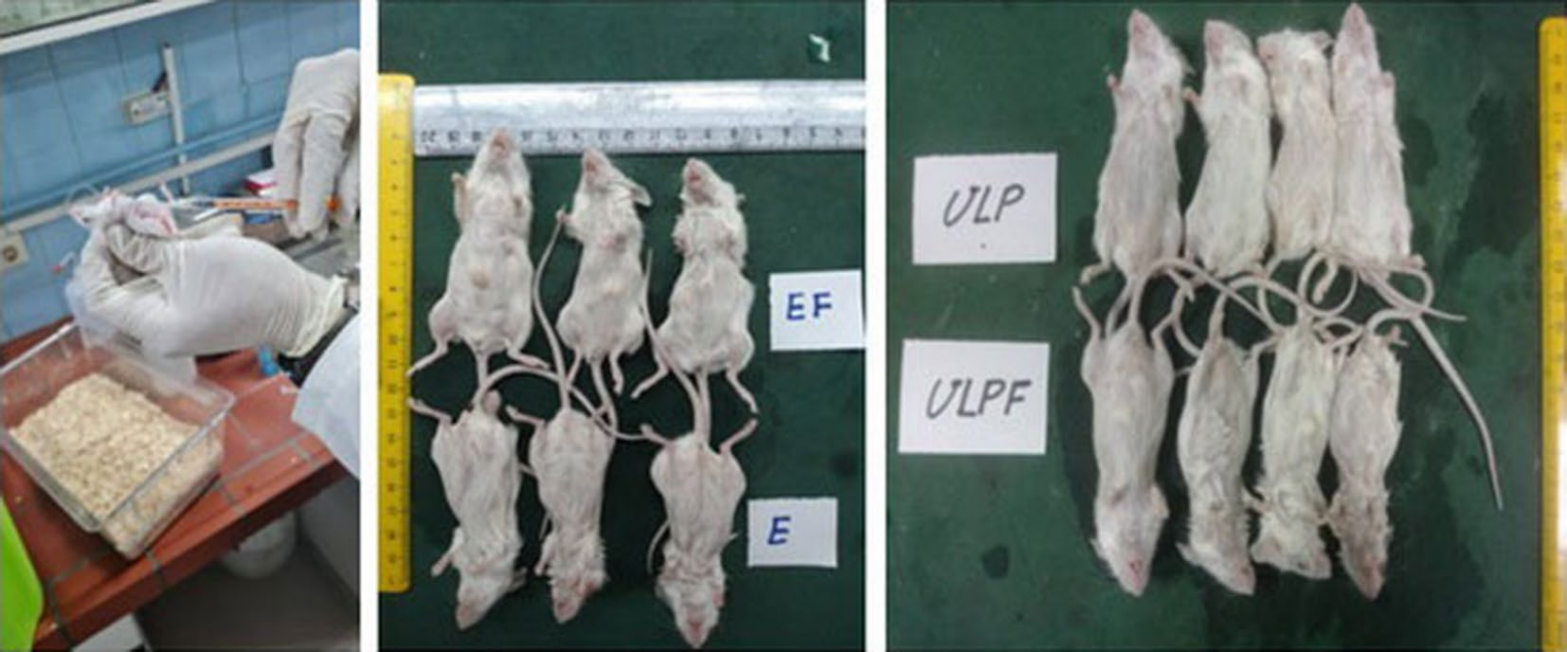
Mice immunization with HBc-VLP-E and HBc-VLP

For the immunization study, pathogen-free female BALB/c mice provided by the Pathobiology Department of Veterinary School, Shiraz University, Iran, aged 6–8 weeks were used. We used three mice for each group and they were immunized separately as different biological repeats two times with two intervals (day 0 and day 14). The mice were injected intramuscularly with 40 μg of HBc-VLP -E and HBc-VLP recombinant proteins (groups 1 and 2). Moreover, to evaluate the effect of the adjuvant, we immunized mice with 40 μg of HBc-VLP -E and HBc-VLP recombinant proteins plus 100 μl of Freund’s adjuvant in each group (groups 3 and 4) (Fig. 4). For groups immunized by recombinant proteins plus adjuvant, Freund’s complete adjuvant and Freund’s incomplete adjuvant were applied for the first and the second injections, respectively. In the negative control group, the mice received 100 μL of PBS buffer (group 5). Serum sample collection was done two and four weeks following the second injection for ELISA analysis. For serum collection, the blood samples were kept at room temperature for one hour, and the serums were separated through centrifugation at 4000 g for 10 min at 4 °C. The serum samples were kept at − 20 °C for the next analysis.

### Immunogenicity analysis

To explore the IgG antibody against HBc-VLP-E that included the epitope of SARS-CoV-2 spike protein, an ELISA test was carried out. Serum samples collected from mice were diluted 1:50 with sterile water, and 100 μl of diluted serum samples were added to the ELISA plates coated by specific SARS-CoV-2 spike antigen (Pishtazteb Company, Iran). After incubation and washing processes with PBST buffer, 100 μl of 1:1000 diluted anti-mouse IgG conjugated with HRP was applied as a secondary antibody per well. Following the incubation and washing, the 3,3’,5,5’-tetramethylbenzidine (TMB) substrate for horseradish peroxidase was added, kept in dark for 15 min, and then the reactions were stopped by 2 M sulfuric acid. Finally, the absorbance was measured at 450 nm by the ELISA reader.

### Statistical analyses

The normality of the data was checked based on the Shapiro–Wilk Test. (α = 0.05). Immunogenicity data generated by the ELISA test were analyzed by Duncan’s test using the Agricola package in R to indicate the significant differences between the quantified IgG antibodies in serum samples collected from HBc VLP, HBc VLP-E, and PBS injections at a 99% confidence interval. The assessments were represented in the graph as an average of three repeats with their standard deviation (SD).

## Data Availability

The datasets generated during and/or analyzed during the current study are available from the corresponding authors upon reasonable request.

## Abbreviations

VLP: Virus-like particle
TEM: Transmission electron microscopy
MIR: Major immunodominant region
ACE2: Angiotensin-converting enzyme 2

## References

[1] Krammer F (2020). SARS-CoV-2 vaccines in development. Nature.

[2] Chang CW, Parsi KM, Somasundaran M, Vanderleeden E, Liu P, Cruz J, Cousineau A, Finberg RW, Kurt-Jones EA (2022). A newly engineered A549 cell line expressing ACE2 and TMPRSS2 is highly permissive to SARS-CoV-2, including the delta and omicron variants. Viruses.

[3] Naz A, Shahid F, Butt TT, Awan FM, Ali A, Malik A (2020). Designing multi-epitope vaccines to combat emerging coronavirus disease 2019 (COVID-19) by employing immuno-informatics approach. Front Immunol.

[4] Mollarasouli F, Zare-Shehneh N, Ghaedi M (2022). A review on corona virus disease 2019 (COVID-19): current progress, clinical features and bioanalytical diagnostic methods. Microchim Acta.

[5] Walsh EE, Frenck RWJ, Falsey AR, Kitchin N, Absalon J, Gurtman A, Lockhart S, Neuzil K, Mulligan MJ, Bailey R (2020). Safety and immunogenicity of two rna-based Covid-19 vaccine candidates N. Engl. J. Med.

[6] Folegatti PM, Ewer KJ, Aley PK, Angus B, Becker S, BelijRammerstorfer S, Bellamy D, Bibi S, Bittaye M, Clutterbuck EA (2020). Safety and immunogenicity of the ChAdOx1 nCoV-19 vaccine against SARS-CoV-2: a preliminary report of a phase 1/2, single-blind, randomised controlled trial. Lancet.

[7] Rohovie MJ, Nagasawa M, Swartz JR (2017). Virus-like particles: Next-generation nanoparticles for targeted therapeutic delivery. Bioeng Transl Med.

[8] Guo J, Zhou A, Sun X, Sha W, Ai K, Pan G, Zhou C, Zhou H, Cong H, He S (2019). Immunogenicity of a virus-like-particle vaccine containing multiple antigenic epitopes of *toxoplasma gondii* against acute and chronic toxoplasmosis in mice. Front Immunol.

[9] Glasgow J, Tullman-Ercek D (2014). Production and applications of engineered viral capsids. Appl Microbiol Biotechnol.

[10] Rodríguez-Limas WA, Sekar K, Tyo KE (2013). Virus-like particles: the future of microbial factories and cell-free systems as platforms for vaccine development. Curr Opin Biotechnol.

[11] Qu Y, Zhang B, Wang Y, Yin S, Sun Y, Middelberg A, Bi J (2021). Immunogenicity and vaccine efficacy boosted by engineering human heavy chain ferritin and chimeric hepatitis b virus core nanoparticles. ACS Appl Bio Mater.

[12] Lei Y, Shao J, Zhao F, Li Y, Lei C, Ma F, Chang H, Zhang Y (2019). Artificially designed hepatitis B virus core particles composed of multiple epitopes of type A and O foot-and-mouth disease virus as a bivalent vaccine candidate. J Med Virol.

[13] Hou Y, Yan T, Cao H, Liu P, Zheng K, Li Z, Deng Q, Hu S (2019). Chimeric hepatitis B virus core particles displaying Neisserial surface protein A confer protection against virulent Neisseria meningitidis serogroup B in BALB/c mice. Int J Nanomed.

[14] Wang YS, Ouyang W, Liu XJ, He KW, Yu SQ, Zhang HB, Fan HJ, Lu CP (2012). Virus-like particles of hepatitis B virus core protein containing five mimotopes of infectious bursal disease virus (IBDV) protect chickens against IBDV. Vaccine.

[15] Sun X, Xing S, Wang S, Zhang X, Yu Y, Wang L (2022). In vitro assembly of chimeric virus-like particles composed of a porcine circovirus 2b capsid protein and a B-cell epitope of infectious bursal disease virus. Biotech Lett.

[16] Pumpens P, Grens E (1999). Hepatitis B core particles as a universal display model: a structure-function basis for development. FEBS Lett.

[17] Pumpens P, Grens E (2001). HBV core particles as a carrier for B cell/T cell epitopes. Intervirology.

[18] Baets SD, Roose K, Schepens B, Saelens X, Buonaguro FM, Buonaguro L (2014). Presenting heterologous epitopes with hepatitis B core-based virus-like particles. Virus-Like Particles in Vaccine Development.

[19] Roose K, Baets SD, Schepens B, Saelens X (2013). Hepatitis B core–based virus–like particles to present heterologous epitopes. Expert Rev Vaccines.

[20] Middelberg AP, Rivera-Hernandez T, Wibowo N, Lua LH, Fan Y, Magor G, Chang C, Chuan YP, Good MF, Batzloff MR (2011). A microbial platform for rapid and lowcost virus-like particle and capsomere vaccines. Vaccine.

[21] Liew MW, Rajendran A, Middelberg AP (2010). Microbial production of virus-like particle vaccine protein at gram-per-litre levels. J Biotechnol.

[22] Yong CY, Liew WPP, Ong HK, Poh CL (2022). Development of virus-like particles-based vaccines against coronaviruses. Biotechnol Prog.

[23] Kaewborisuth C, Wanitchang A, Koonpaew S, Srisutthisamphan K, Saenboonrueng J, Im-Erbsin R, Inthawong M, Sunyakumthorn P, Thaweerattanasinp T, Tanwattana N, Jantraphakorn Y (2022). Chimeric virus-like particle-based COVID-19 vaccine confers strong protection against SARS-CoV-2 viremia in K18-hACE2 mice. Vaccines.

[24] Wong TY, Russ BP, Lee KS, Miller OA, Kang J, Cooper M, Winters MT, Rodriguez-Aponte SA, Dalvie NC, Johnston RS, Rader NA (2022). RBD-VLP vaccines adjuvanted with alum or SWE protect K18-hACE2 mice against SARS-CoV-2 VOC challenge. Msphere.

[25] Ghorbani A, Zare F, Sazegari S, Afsharifar A, Eskandari MH, Pormohammad A (2020). Development of a novel platform of virus-like particle (VLP)-based vaccine against COVID-19 by exposing epitopes: an immunoinformatics approach. New Microbes New Infect.

[26] Linda N, Lua Frank Sainsbury HL, Yap P, Wibowo CN, Middelberg APJ (2014). Bioengineering virus-like particles as vaccines. Biotechnol Bioeng.

[27] Yan D, Wei YQ, Guo HC, Sun SQ (2015). The application of virus-like particles as vaccines and biological vehicles. Appl Microbiol Biotechnol.

[28] Blokhina EA, Kuprianov VV, Stepanova LA, Tsybalova LM, Kiselev OI, Ravin NV, Skryabin KG (2013). A molecular assembly system for presentation of antigens on the surface of HBc virus-like particles. Virology.

[29] Peyret H, Gehin A, Thuenemann EC, Blond D, El Turabi A, Beales L, Clarke D, Gilbert RJ, Fry EE, Stuart DI, Holmes K (2015). Tandem fusion of hepatitis B core antigen allows assembly of virus-like particles in bacteria and plants with enhanced capacity to accommodate foreign proteins. PLoS ONE.

[30] Sani MZ, Bargahi A, Momenzadeh N, Dehghani P, Moghadam MV, Maleki SJ, Nabipour I, Shirkani A, Akhtari J, Hesamizadeh K, Heidari S (2021). Genetically engineered fusion of allergen and viral-like particle induces a more effective allergen-specific immune response than a combination of them. Appl Microbiol Biotechnol.

[31] Lei Y, Shao J, Zhao F, Li Y, Lei C, Ma F, Chang H, Zhang Y (2019). Artificially designed hepatitis B virus core particles composed of multiple epitopes of type A and O foot-and mouth disease virus as a bivalent vaccine candidate. J Med Virol.

[32] Aston-Deaville S, Carlsson E, Saleem M, Thistlethwaite A, Chan H, Maharjan S, Facchetti A, Feavers IM, Siebert CA, Collins RF, Roseman A (2020). An assessment of the use of Hepatitis B virus core protein virus-like particles to display heterologous antigens from *Neisseria* meningitidis. Vaccine.

[33] Ye X, Ku Z, Liu Q, Wang X, Shi J, Zhang Y, Kong L, Cong Y, Huang Z (2014). Chimeric virus-like particle vaccines displaying conserved enterovirus 71 epitopes elicit protective neutralizing antibodies in mice through divergent mechanisms. J Virol.

[34] Liang P, Yao YI, Su QD, Feng QIU, Fan XT, Lu XX, Bi SL (2018). Efficient humoral and cellular immune responses induced by a chimeric virus-like particle displaying the epitope of EV71 without adjuvant. Biomed Environ Sci.

[35] Qiao L, Zhang Y, Chai F, Tan Y, Huo C, Pan Z (2016). Chimeric virus-like particles containing a conserved region of the G protein in combination with a single peptide of the M2 protein confer protection against respiratory syncytial virus infection. Antiviral Res.

[36] Huynh NH, Davey K, Jin B, Bi J (2022). A statistical approach to boost soluble expression of *E. coli* derived virus-like particles in shake-flask cultivation. J Biotechnol.

[37] Zeltins A (2013). Construction and Characterization of Virus-Like Particles: A Review. Mol Biotechnol.

[38] Huang X, Wang X, Zhang J, Xia N, Zhao Q (2017). Escherichia coli-derived virus-like particles in vaccine development. npj Vaccines.

[39] Lua LH, Connors NK, Sainsbury F, Chuan YP, Wibowo N, Middelberg AP (2014). Bioengineering virus-like particles as vaccines. Biotechnol Bioeng.

[40] Nooraei S, Bahrulolum H, Hoseini ZS, Katalani C, Hajizade A, Easton AJ, Ahmadian G (2021). Virus-like particles: Preparation, immunogenicity and their roles as nanovaccines and drug nanocarriers. J Nanobiotechnol.

[41] Birkett A, Lyons K, Schmidt A, Boyd D, Oliveira GA, Siddique A, Nussenzweig R, Calvo-Calle JM, Nardin E (2002). A modified hepatitis B virus core particle containing multiple epitopes of the Plasmodium falciparum circumsporozoite protein provides a highly immunogenic malaria vaccine in preclinical analyses in rodent and primate hosts. Infect Immun.

[42] Oliveira GA, Wetzel K, Calvo-Calle JM, Nussenzweig R, Schmidt A, Birkett A, Dubovsky F, Tierney E, Gleiter CH, Boehmer G, Luty AJ (2005). Safety and enhanced immunogenicity of a hepatitis B core particle *Plasmodium* falciparum malaria vaccine formulated in adjuvant montanide ISA 720 in a phase I trial. Infect Immun.

[43] Park MH, You JW, Kim HJ, Kim HJ (2019). IgG and IgM responses to human papillomavirus L1 virus-like particle as a function of dosing schedule and vaccine formulation. J Microbiol.

[44] Liu J, Zhang P, Chen Y, Zhong W, Li B, Pi M, Ning Z (2021). Vaccination with virus-like particles of atypical porcine pestivirus inhibits virus replication in tissues of BALB/c mice. Adv Virol.

[45] Conner ME, Zarley CD, Hu B, Parsons S, Drabinski D, Greiner S, Smith R, Jiang B, Corsaro B, Madore HP, Crawford S (1996). Virus-like particles as a rotavirus subunit vaccine. J Infect Dis.

[46] Skrastina D, Petrovskis I, Lieknina I, Bogans J, Renhofa R, Ose V, Dishlers A, Dekhtyar Y, Pumpens P (2014). Silica nanoparticles as the adjuvant for the immunization of mice using hepatitis B core virus-like particles. PLoS ONE.

[47] Chang X, Zeltins A, Mohsen MO, Gharailoo Z, Zha L, Liu X, Walton S, Vogel M, Bachmann MF (2021). A novel double mosaic virus-like particle-based vaccine against SARS-CoV-2 incorporates both receptor binding motif (RBM) and fusion domain. Vaccines.

[48] Bin Mohamed Suffian IF, Garcia-Maya M, Brown P, Bui T, Nishimura Y, Palermo ARBMJ, Ogino C, Kondo A, Al-Jamal KT (2017). Yield optimisation of Hepatitis B virus core particles in *E. coli* expression system for drug delivery applications. Sci Rep.

[49] Hsieh PK, Chang SC, Huang CC, Lee TT, Hsiao CW, Kou YH, Chen IY, Chang CK, Huang TH, Chang MF (2005). Assembly of severe acute respiratory syndrome coronavirus RNA packaging signal into virus-like particles is nucleocapsid dependent. J Virol.

[50] Mahony JB, Chernesky MA (1991). Negative staining in the detection of viruses in clinical specimens. Micron Microscopica Acta.

